# Two Important Metropolitan Hospitals

**Published:** 1907-07-06

**Authors:** 


### TWO IMPORTANT METROPOLITAN HOSPITALS.

### University College Hospital.

This hospital having been rebuilt and modernised, should, in our judgment, take a great step forward by a selection of the best literary and financial brains amongst the Governors, with the object of reconstructing its present financial position. What is wanted is a material change in the methods of action in regard to appeals and literature, which are in many respects quite out of date. It is no use, we are glad to know, in the present day, to continually go to the public with the cry that if more money is not forthcoming certain wards will have to be closed. The public has become so much more enlightened in recent years that it realises that no committee of a great hospital having any claim to be regarded as a business organisation will put down beds on the ground that by so doing they can materially reduce expenses. The shutting up of fifty or one hundred beds in a great hospital will not represent in the year's accounts a saving of expenditure equal to the actual cost of one hundred occupied beds during the previous twelve months. On the contrary, it is very probable that the reduction in fact will not amount to half such a sum, and this knowledge prejudices the giving public against any charitable institution which puts forward a statement of this kind in support of an appeal for further funds. Again, appeal literature nowadays should be made interesting. It is not necessary to write a volume in order to make out a case, but it is essential that typographically, and in a literary sense, all hospital literature to prove effective must be made attractive and interesting. Can any writer desire a more inviting topic than the work of a modern hospital from day to day throughout the year? We doubt it, for we know that a visit to any one of the great hospitals by an expert always brings out some feature of interest which is novel and attractive to the visitor, though he may have had forty years' experience as an inspector of these institutions. University College Hospital is entitled to the warm and generous support of Londoners, and indeed of moneyed people all over the country. It has rendered great services to all classes in the past, its history is full of interest and instruction, and its connection with University College entitles it to the generous support of all who have money to spare and hearts to sympathise with the relief of sickness and disease. We have therefore thought that the best service we could render to this institution, which has languished financially for so many years, is to plainly state that the remedy will be found in the application of new and modern methods on business lines to the financial arrangements and the raising of revenue. Fortunately it is becoming the universal practice to pension hospital officials, so that most of the difficulties formerly presented when radical changes were called for have now disappeared.

### King's College Hospital.

The sixty-eighth report of this hospital is a business-like document, well and clearly printed, and arranged in a novel way. It commences with a subscriber's order form on the bankers, with a polite intimation that annual subscribers are most needed and most acceptable. Then we have the contents; then regulations as to the admission of and visitors to patients, and the form of bequest. Then the personnel of the management and medical staff, and then the conditions governing endowed wards and beds. As to the last we consider that £1,000 to endow a bed, and £500 to endow a cot are amounts which have been fixed far too low, considering that the earning power of such capital sums is less than half the actual expenditure on an occupied bed or cot each year. The report indicates that there has been a decrease compared with previous years in the work of the out-patient department under practically all heads. We congratulate the management on this fact, which constitutes a strong reason why intelligent people should support King's College Hospital and its present management. The character of the neighbourhood has changed, but the work of the almoners' department has proved effective too we are glad to note, and the department is working in very friendly co-operation with private practitioners, six hundred patients having been sent to the hospital by the latter during the year for consultations and private treatment. It is a sound financial reason for removing the hospital to the suburbs that the recent re-assessment has increased the annual expenditure due to rates and taxes by £216. It is pleasant to note that the committee record their thanks to King Edward's Hospital Fund for the statistical returns which have helped to promote economy in the working of the hospital. The services of the late Mr. J. H. Saunders, for thirty-five years steward and clerk of the hospital, are cordially recognised, and it is pleasant to note that the vacancy thus caused will be filled by the promotion of Mr. H. Cheffings, who has been in the service of the hospital for twenty-one years. This hospital needs, deserves and should receive the support of the most intelligent members of that large class of the community who select hospitals as the channel for their charitable gifts. At the annual dinner on Friday the 28th ultimo Mr. Watson Cheyne, one of Lord Lister's house surgeons, naturally and deservedly received grateful recognition. The hospital has very substantial friends, and amongst them Mr. W. F. D. Smith, whose gift of a new site is valued at £62,000. We quite agree with the chairman that the time is far distant when the present voluntary system of hospital support will be superseded, and that, if ever that time should come, the ratepayers will never fail to regret it. It was disappointing that the dinner resulted in a contribution of only £1,400, but the times, as we said last week, are exceptional, and festival dinners are not capable of producing, at the moment, the large sums they have sometimes yielded in the past.

